# P-912. Correlation between Novel Antibiotic Stewardship Metrics and Antibiotic Use Practices for Lower Respiratory and Urinary Tract Infection across 24 VA Hospital Systems

**DOI:** 10.1093/ofid/ofaf695.1118

**Published:** 2026-01-11

**Authors:** Thomas Wright, Daniel J Livorsi, James Merchant, Hyunkeun Cho, Christopher Richards, Brice Beck, Bruce Alexander, Michihiko Goto

**Affiliations:** University of Iowa Carver College of Medicine, Fort Wayne, IN; University of Iowa Carver College of Medicine, Fort Wayne, IN; Iowa City VA Health Care System, Iowa City, Iowa; University of Iowa Carver College of Medicine, Fort Wayne, IN; Iowa City VA Health Care System, Iowa City, Iowa; Iowa City VA Health Care System, Iowa City, Iowa; Iowa City VA Medical Center, Iowa City, Iowa; University of Iowa/Iowa City VAMC, Iowa City, IA

## Abstract

**Background:**

Various metrics have been proposed to measure antibiotic consumption in inpatient settings, but little is known about how these metrics correlate with appropriateness of antibiotic usage (i.e. construct validity). We developed two metrics (risk-standardized ratio: RSR), one based on Days of Therapy (RSR-DOT) and another based on Days of Antimicrobial Spectrum Coverage (RSR-DASC). We aimed to evaluate whether hospital performance on these metrics is associated with appropriateness of antibiotic selection and duration.

**Table 1:**
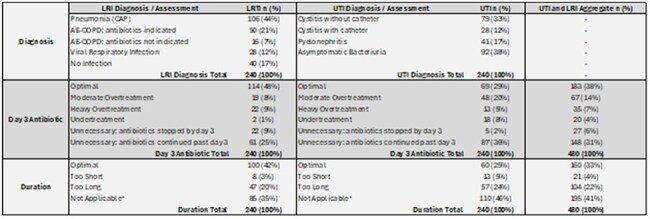
Assessments of Diagnosis, Antibiotic Selection on Day 3, and Antibiotic Duration for LRTI and UTI

**Figure 1:**
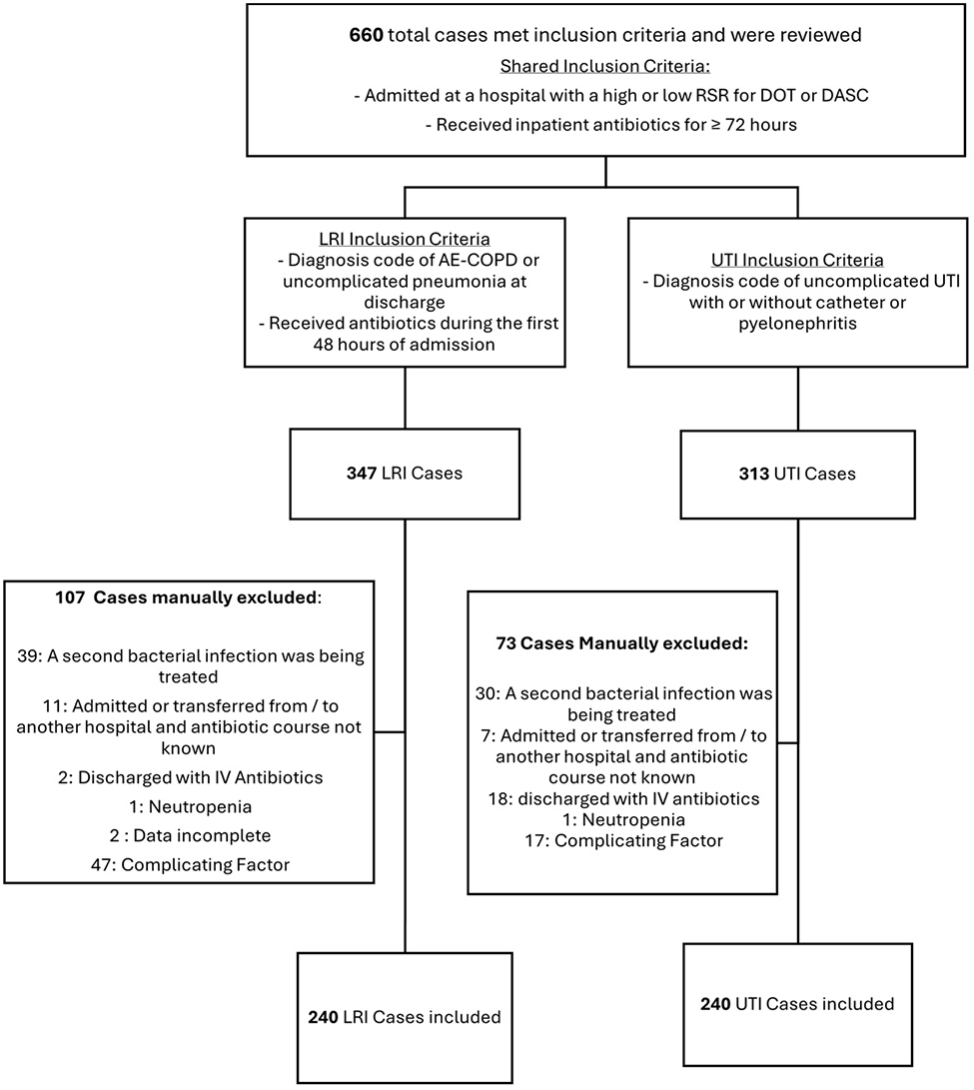
Application of Inclusion and Exclusion Criteria

**Methods:**

Using data from October 2020 to September 2021, we constructed RSR-DOT and RSR-DASC for 118 acute-care hospitals in the Veterans Health Administration and selected 24 with lower or higher RSRs for DOT and DASC. For each hospital, an Infectious Disease physician, blinded to the hospital’s performance, reviewed 10 cases each of lower respiratory tract infections (LRI) and urinary tract infections (UTIs) to assess the appropriateness of antibiotic selection on day 3 (6-level ordinal outcomes) and appropriateness of antibiotic duration. Associations with RSR metrics were assessed using ordinal logistic regression (antibiotic selection) and logistic regression (antibiotic duration).

**Figure 2:**
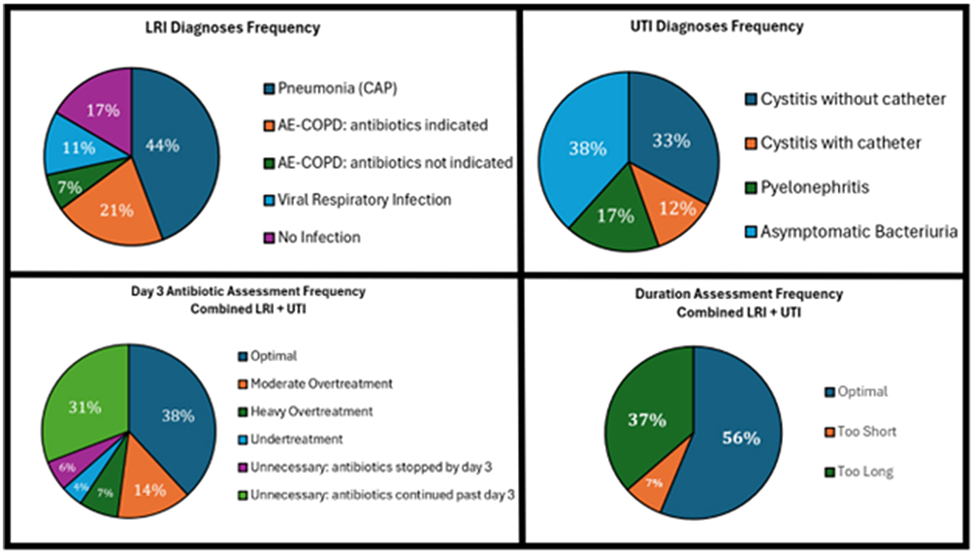
Pie Charts representing Frequency of LRTI and UTI Diagnosis, Antibiotic Selection on Day 3, and Duration Assessment

**Table 2.**
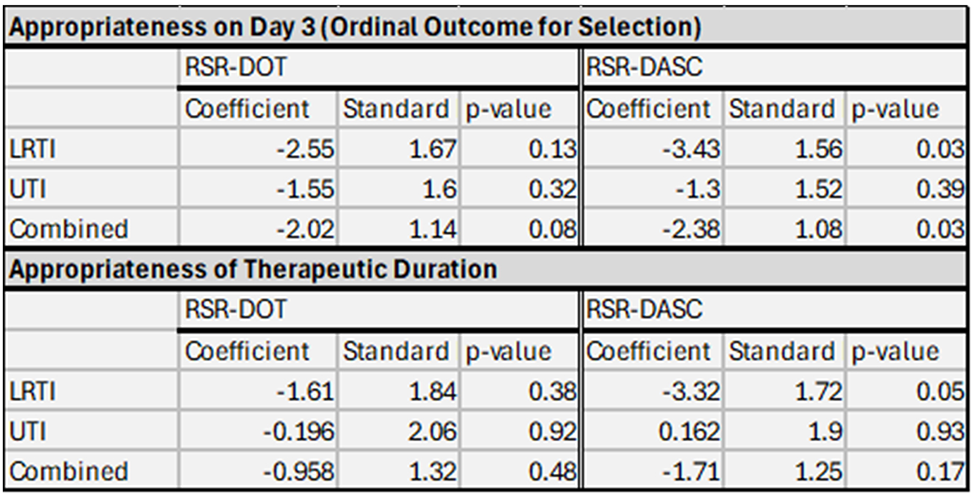
Association between Hospital Performance on the risk-standardized metrics and antibiotic appropriateness for LRTI and UTI

**Results:**

The final cohort included 240 cases for both LRI and UTI diagnoses after applying exclusion criteria (Figure 1 and Table 1). Lower RSR-DASC was significantly associated with better antibiotic selection at day 3 and appropriate duration for LRI, while RSR-DOT did not show significant associations (Table 2). For UTIs, we did not observe significant associations between antibiotic selection at day 3 or appropriate duration with either metric. When cases of LRI and UTI were combined, lower RSR-DASC was significantly associated with better antibiotic selection at day 3.

**Conclusion:**

Hospital evaluations by RSR-DASC were associated with appropriateness of antibiotic selection at day 3 and the antibiotic duration in LRI, while RSR-DOT did not show any significant associations, suggesting higher construct validity with RSR-DASC. The lack of associations with both metrics in UTI might be due to fewer intra-hospital differences in antibiotic selection when microbiologic data (e.g. urine cultures) are available and high frequency of asymptomatic bacteriuria treatment.

**Disclosures:**

All Authors: No reported disclosures

